# Review on allosteric modulators of dopamine receptors so far

**DOI:** 10.1002/hsr2.1984

**Published:** 2024-03-18

**Authors:** Fentaw Girmaw

**Affiliations:** ^1^ Department of Pharmacy, College of Health Science Woldia University Woldia Ethiopia

**Keywords:** allosteric, dopamine, modulators, receptors

## Abstract

Background

Contemporary research is predominantly directed towards allosteric modulators, a class of compounds designed to interact with specific sites distinct from the orthosteric site on G protein‐coupled receptors. These allosteric modulators play a pivotal role in influencing diverse pharmacological effects, such as agonism/inverse agonism, efficacy modulation, and affinity modulation. One particularly intriguing aspect is the demonstrated capacity of allosteric modulation to enhance drug selectivity for therapeutic purposes, potentially leading to a reduction in serious side effects associated with traditional approaches. Allosteric ligands, a majority of which fall into the categories of negative allosteric modulators or positive allosteric modulators, exhibit the unique ability to either diminish or enhance the effects of endogenous ligands. Negative allosteric modulators weaken the response, while positive allosteric modulators intensify it. Additionally, silent allosteric modulators represent a distinct class that neither activates nor blocks the effects of endogenous ligands, adding complexity to the spectrum of allosteric modulation. In the broader context of central nervous system disorders, allosteric modulation takes center stage, particularly in the realm of dopamine receptors specifically, D1, D2, and D3 receptors. These receptors hold immense therapeutic potential for a range of conditions spanning neurodegenerative disorders to neurobehavioral and psychiatric disorders. The intricate modulation of dopamine receptors through allosteric mechanisms offers a nuanced and versatile approach to drug development. As research endeavors continue to unfold, the exploration of allosteric modulation stands as a promising frontier, holding the potential to reshape the landscape of drug discovery and therapeutic interventions in the field of neurology and psychiatry.

## 1 INTRODUCTION

Traditional drug discovery approaches targeting G‐protein‐coupled receptors (GPCRs) have historically focused on developing ligands for orthosteric sites. To activate or inhibit receptor response, allosteric modulators target allosteric sites, which differ from orthosteric sites is a recent concern.^1^ This creates a new therapeutic target for a number of CNS disorders, including neurodegenerative, neurobehavioral, and psychiatric disorders. As allosteric modulators for GPCRs, endogenous molecules like lipids, peptides, and ions can also function. The interaction between ligands and GPCRs is significantly impacted by the binding of ions like Ca2+, Na+, and Zn2+.^1^, ^2^, ^3^, ^4^ GPCRs, also known as seven transmembrane spanning receptors, are the largest family of cell surface receptors.^3^

In order for dopamine to have an effect, it needs to attach itself to specific receptors located on the outer membrane of target cells. These receptors belong to a group of proteins called G protein‐coupled receptors (GPCRs). The existence of dopamine receptors was first discovered in 1972, and they were identified in 1975.^5^, ^6^ There are currently five known subtypes of dopamine receptors: D1, D2, D3, D4, and D5. All of these receptors work through a process called metabotropic signaling, which involves the activation of second messengers. These second messengers then either stimulate or inhibit specific cell signaling pathways.^6^, ^7^

Based on their structure and effects on the body, dopamine GPCR receptors can be categorized into two main groups: D1‐like receptors, which include D1 and D5; and D2‐like receptors, which consist of D2, D3, and D4.^7^, ^8^ Dopamine receptors are found throughout the central nervous system and also in various peripheral organs such as blood vessels, kidneys, heart, retina, and adrenals. In the brain, the D1 and D2 receptors are the most commonly found dopamine receptors, with D1 being the most abundant. It is uncommon for these two receptor types to be present in the same cells. These receptors play a role in regulating the release of catecholamine and the renin‐angiotensin system.^8^ D1 and D5 receptors activate Gαs, olf protein, leading to an increase in the production of a molecule called cAMP. In contrast, D2, D3, and D4 receptors activate Gαi/o protein, which reduces the production of cAMP within the cell.^9^

Many commercially available drugs directly activate or block signaling cascades that are mediated by these receptors. However, due to the high degree of homology in the ligand‐binding site between GPCR subtypes such as dopamine D2 and dopamine D3, it has proven difficult to develop small molecules for some GPCRs such as peptide receptors and difficult to achieve sufficient selectivity for others.^3^, ^4^ After binding with their respective allosteric sites, the associations between an allosteric ligand and a GPCRs lead to various conformational changes.^10^

Surprisingly, the binding of an allosteric modulator to a receptor can result in a variety of pharmacological effects such as modulation of affinity, efficacy, and agonism/inverse agonism, whether there is an orthosteric ligand present or not.^11^, ^12^ In fact, the discovery of G‐protein‐coupled receptor (GPCR) allosteric modulators has garnered a lot of attention for its potential as a therapeutic agent and a tool for understanding receptor mechanisms. The identification of small molecules that target sites different from the orthosteric natural agonist and that cause a conformational change in the GPCR, thereby allosterically modulating the receptor function, has therefore become a major focus of drug research.^13^ As a result, the allosteric modulators can present implicit advantages from a medicine‐discovery perspective. First, it lacks direct effect that only potentiate the effect of the native transmitter where and when it is released. Second, it can reduce propensity for inducing desensitization arising from the constant exposure to an agonist. Third, it can also increase subtype selectivity that prevents overdosing. Fourth, it introduces specific useful pharmacological effects without affecting the integrity of complex physiologically regulated networks.^10^, ^14^

Since allosteric modulators are extremely sensitive to changes in protein conformation, they have been used to determine whether a specific mutation results a global change in protein conformation.^15^ Allosteric ligands can be categorized into three groups based on their pharmacological effects: positive allosteric modulators (PAMs), negative allosteric modulators (NAMs), and silent allosteric modulators (SAMs). At dopamine receptors, all the three types of allosteric modulation are possible.^16^, ^17^, ^18^ These allosteric modulators can affect affinity and/or efficacy to mimic or attenuate the effects of the endogenous ligand.^3^, ^19^ Furthermore, SAMs are additional compounds that bind to the allosteric site but do not activate or inhibit responses.^11^, ^20^, ^21^

## 2 METHODS

The literature to this narrative review was extracted through electronic search from PubMed, EMBASE, Google Scholar, Scopus, Medline, and Manual search. Full text publications were reviewed for potential inclusion but publications without methods and content redundant were excluded.

### 2.1 Allosteric modulators of dopamine receptors

Dopamine receptors are under the class A family of GPCRs that are considered as a therapeutic target for treatment of CNS and non‐CNS disease. This is because of their capability to modulate cellular responses to a neurotransmitter dopamine with several important functions in the CNS and the PNS.^22^ Dopamine works through its respective GPCRs to activate or attenuate reward mechanisms, motor function, central processing, and other physiologic activities.^22^, ^23^

Based on the molecular characteristic's dopamine receptors are classified primarily as D1‐like and D2‐Like receptors. D1‐like receptors are further categorized into D1 and D5 receptors while D2‐Like receptors are categorized into D2 (D2S and D2L), D3, and D4 receptors. All these five receptor subtypes are activated by endogenous catecholamine called dopamine. Members of the same class share considerable similarities. The D1‐like (D1 and D5) receptors share an 80% identity in their transmembrane domains. However, in D2‐like receptors the D2 and D3 receptors have a 75%, and the D2 and D4 receptors have a 53% shared identity in the transmembrane domains.^23^, ^24^, ^25^, ^26^ From these five dopamine receptors, D1 and D2 are the most widely studied subtypes due to their significant functions in reward, motor activity and their greater expression level.^26^, ^27^, ^28^

Although some current marketed dopaminergic drugs potentially have D1 activity,^29^, ^30^ utmost available medicines substantially target D2/D3 receptors. The first generation of D1 orthosteric agonizts has been limited to invasive therapies. The main reason for this is associated with their poor CNS penetration and low oral bioavailability which is related with the highly polar catechol moiety of dopamine.^31^ Indeed, orthosteric ligand binding to dopamine receptor for dopamine mediated signaling pathway is critical. However, the activation or inhibition of these signaling pathways can be modulated by allosteric ligands binding to their allosteric site. Like other GPCRs ions such as Zn2+ can also modulate the orthosteric ligand activities at D1, D2 and D4 dopamine receptors allosterically.^3^, ^4^

### 2.2 Allosteric modulators of D_1_ receptors

The dopamine D1 receptor is the most widely expressed receptor among the five dopamine receptor subtypes. Even though clinically useful selective ligands for D1 receptor are rare, the first dopamine D1 receptor selective ligand was introduced three decades back.^32^ The benzazepines and the non‐benzazepines are the two series of classical D1 orthosteric ligands, which bind to the dopamine D1 receptor.^32^, ^33^ Most orthosteric agonizts that are available in recent time were predominantly of catecholamine origin.^32^ Clinical developments of these agonizts has been very difficult and largely unsuccessful so far due to a combination of chemical and pharmacological challenges. Chemically, catecholamine agonizts are prone to air oxidation, leading to manufacturing and storage issues. In addition, they also display poor bioavailability and short t_1/2_.^25^ Pharmacologically, these agonizts have shown also poor selectivity over the other dopamine receptors since they bind to the highly conserved orthosteric site. The earlier discovered D1 orthosteric agonizts had a moderate selectivity over the other dopamine receptors. Even the recent reports indicated that non‐catecholamine D1 partial agonizts do not effectively differentiate between human D1 and D5 subtypes.^34^, ^35^

Furthermore, the catecholamine‐based D1 agonizts produced bell‐shaped dose–response relationship due to the overstimulation at higher doses in animal models of cognition and locomotor activity.^36^, ^37^ Some D1 agonizts have developed tolerance (tachyphylaxis) that result a rapid loss of efficacy. This is related to the constantly activation of all accessible D1 receptors by the agonist for as long as they are present which further introduce a subsequent rapid receptor desensitization.^38^ The dose limiting side effects such as nausea and hypotension have been observed in the clinic for some of these D1 agonizts.^25^

Considering the challenges to develop useful orthosteric D1 agonizts, an alternative strategy that upregulate D1 receptor activity is highly desirable.^39^, ^40^ As a result, the positive allosteric modulation strategy to increase D1 receptor activity is important for the following reasons: First, binding at an allosteric site of the dopamine D1 receptor will likely require a pharmacophore different from the catecholamine, addressing the chemical stability and pharmacological selectivity issues associated with the orthosteric agonizts. Second, the more physiologically relevant mode of action of a D1 PAM may have a potentially better safety profile as well as a lower propensity for overstimulation and tolerance development. However, the human safety profile of a D1 PAM cannot be fully assessed. In the presence of micromolar concentrations of Zn2+ the binding of D1 antagonists to D1 dopamine receptors is reduced allosterically because zinc can increase the dissociation constant of ligand binding.^4^

Studies have disclosed various series of D1 PAMs from several non‐catecholamine chemical scaffolds in the scientific literature, issued patents, and patent applications. In particular, characterization of DETQ, a novel, highly potent, and subtype selective D1 PAM is suitable tool compound to study allosteric activation of the D1 receptor in vivo.^41^, ^42^, ^43^, ^44^ The DETQ and CID 2886111 simultaneously can interact to the dopamine D1 receptor. This combination results supra‐additive response even in the absence of dopamine.^43^ In addition, DETQ improves object recognition memory deficits, improving the cognitive function in the sub‐chronic PCP mouse model. The lack of an inverted U‐shaped dose–response curve with DETQ reduces concern of over stimulating D1Rs in patients with high D1. This makes DETQ more preferred over D1R orthosteric agonizts.^44^

In the current account, the synthesis and pharmacological characterization of LY3154207, which is currently in phase 2 development for the treatment of Lewy Body Dementia can address the challenge in the discovery of orthosteric agonizts.^45^

In preclinical animal models, D_1_ agonizts have been mediated cognitive enhancing effects with an inverted U‐shaped dose–response curve, suggesting that too little or too much D_1_ activation can be detrimental.^37^ However, a proof‐of‐principle study revealed that administration of the D_1_‐preferring agonist dihydrexidine improved working memory in patients with schizotypal personality disorder.^46^ Unfortunately, dihydrexidine, which is only moderately selective for D_1_ over D_5_, has poor bioavailability and is rapidly metabolized which limits the clinical utility of this compound. Furthermore, new D_1_‐selective PAMs have been developed recently with an enhanced specificity over D_5_ receptors.^47^

D_1_ PAMs, due to their mechanism of action, have the potential to avoid the adverse effects seen with excessive D_1_ receptor activation. It also induced changes in locomotion in a humanized mouse line that plateaued at high doses without inducing stereotypies unlike D_1_ agonizts.^40^ The recent discovery D_1_‐selective allosteric scaffolds represent an important advance and hopefully lead to optimized compounds that can provide therapeutically desirable outcomes with fewer side effects than those observed with D1 receptor agonizts and antagonists. Studies also showed a surprising location for a D1 PAM binding site, around intracellular loop‐2 (ICL2) and more than one PAM site may exist in D1 receptor.^31^

### 2.3 Allosteric modulators of D_2_ receptors

The dopamine D_2_ receptor is a key drug target for the treatment of many central nervous system diseases.^48^, ^49^ This receptor has been an important target for Parkinson's disease^50^, ^51^, ^52^ and a therapeutic strategy in Alzheimer's disease.^53^, ^54^ In addition, it is also a focus of typical and atypical antipsychotics which are used for the treatment of schizophrenia.^50^, ^51^

The gene for the dopamine D2 receptor produces D2S and D2L isoforms which have 415 and 444 amino acids in length respectively. In addition, they also contain a 29‐amino‐acid fragment in intracellular loop 3.^55^ The D2S receptors are mainly presynaptic while D2L receptors are mainly postsynaptic. In view of importance the presynaptic receptor (D2S) are auto‐receptors that inhibit neurotransmission in dopaminergic neurons.^56^ In addition, subtle ligand modifications for the D2L receptors can affect its allosteric modulation. As a whole the functional outcome of the allosteric modulator of these receptors are determined by the chiral carbon configuration and/or structural modulator modifications.^16^

The D_2_ receptor can be stimulated by the natural catecholamine neurotransmitter dopamine or by synthetic ligands (orthosteric or allosteric ligands). This leads in to the coupling of receptor with G‐protein which later changes the D2 receptor mediated signaling pathways.^49^ Drug discovery at D2 receptors has primarily focused on targeting the orthosteric site with agonizts and antagonists. The agonizts are used for the treatment of parkinson's disease, and the antagonists for schizophrenia. However, allosteric site targeting of D2 receptors may provide many advantages over orthosteric site such as maintenance of spatiotemporal patterns.^44^

The discovery of the first NAM of the D_2_ receptor (SB269652) displayed a turning point to achieve allosteric modulation of this target less potently.^57^ It adopts a bitopic mode of action, binding to both an allosteric and the orthosteric site. The tetrahydroisoquinoline moiety bind with the orthosteric binding site and the indole‐2‐carboxamide moiety occupies a secondary binding pocket. Here in, the structural changes to indole‐2‐carboxamide motif allows the generation of a novel set of analogs with distinct pharmacological properties with dramatic increase in functional affinity, cooperativity and efficacy. It also demonstrated that the NAM activity of SB269652 is at a functional D_2_ receptor homodimer only.^58^, ^59^, ^60^

Sodium ions (Na+) allosterically modulate the binding of orthosteric agonizts and antagonists to the dopamine D2 receptor. The presence of Na+ within the conserved Na+ binding pocket is required for the action of SB269652 and it is required for the high affinity binding of the tetrahydroisoquinoline moiety within the orthosteric binding site. Furthermore, the association of the indole‐2‐carboxamide moiety with the secondary binding pocket determines the degree of Na+ sensitivity. Considering the synergistic effect of SB269652 and Na+ to modulate the binding of orthosteric ligands at the D2R provides opportunities in future for allosteric modulatory effect drug discovery.^60^, ^61^

The tripeptide, PLG is an endogenous allosteric modulator of D2 receptors which can enhances the binding activity of orthosteric agonizts and promotes a high‐affinity receptor conformation. Due to the minor variation in the stereochemistry of PLG peptidomimetics, a small structural alteration in the spiro‐bicyclic dopamine receptor modulators causes a dramatic change in their modulatory activities.^62^, ^63^ PLG has also demonstrated a pharmacological promise in clinical studies of neuropsychiatric disease even in major depressive disorder.^63^, ^64^

From more than 200 analog molecules of PLG, PAOPA has showed more potent positive allosteric activity for the dopamine D2 receptors.^65^ It's binding with the dopamine D2 receptor has been well characterized^66^ and it has attenuated biochemical and behavioral abnormalities specific to the positive ‐and negative‐like symptoms of schizophrenia in preclinical models.^67^ PAOPA also displayed remarkable effects in treating and preventing akin to the negative‐like symptoms, amphetamine‐induced sensorimotor gating deficits and akin to the positive‐like symptoms.^68^, ^69^ Since all available antipsychotics in the market act via downregulation of D_2_ signaling, a D_2_‐selective NAM or partial NAM that provide antipsychotic efficacy with reduced motor and cognitive side effects and selectively modulates only certain D_2_ dimer complexes can minimize the problem.

Allosteric modulation of certain ligands can also occur at the level of receptor heterodimers. Previous studies revealed that a dopamine agonist can negatively modulate adenosine A_2A_ receptor binding properties by interacting with the A_2A_–D_2_ receptor heteromers.^70^ Interestingly, A_2A_ receptor agonist induced modulation of D_2_ receptor agonist‐induced βArr2 recruitment has been suggesting the involvement of a possible A_2A_–D_2_–βArr2 complex in this allosteric modulation.^71^ Both A_2A_ receptor agonizts and antagonists decrease the affinity and intrinsic efficacy of D_2_ receptor agonizts and the affinity of D_2_ receptor antagonists. However, on agonist and antagonist coadministration these allosteric modulations are disappeared.^72^

### 2.4 Allosteric modulators of D_3_ receptors

Dopamine D_3_ receptors are D_2_‐like family of GPCRs that signal via the Gα_i_ to inhibit adenylyl cyclase (AC) and activate various kinase signaling pathways.^73^ They involved in the control of mood, cognition, and motor behaviors.^74^ They are also a target to manage drug addiction and schizophrenia due to their enrichment in mesolimbic dopaminergic projection areas.^14^, ^22^, ^75^, ^76^ Based on the crystal structure of D_3_ receptors^77^ a virtual screen targeting allosteric sites of this receptor^78^ resulted in the identification of a number of allosteric ligands. It includes a chemically diverse compounds with a variety of functional activity profiles and high affinities. It is believed that knowing the allosteric structural features of D_3_ receptors that are essential for selectivity and efficacy could be critical in the identification of highly selective D_3_ receptor ligands.^67^, ^68^, ^79^

Agonizts at dopamine D3 receptors are important therapeutic agents in the treatment of parkinson's disease. Allosteric potentiator offer potential advantages over the use of agonizts. It includes phasic, temporal, and regional potentiation of natural signaling, and that of receptor subtype selectivity.^80^ The stereoselective interaction of a benzothiazol racemic compound which is a PAM of D3 receptors demonstrated that the R isomer did not directly stimulate the dopamine D3 receptor but potentiated the effects of dopamine. However, the S isomer block the effects of the dopamine and PAM. These compounds do not compete for binding of orthosteric ligands, but the R isomer increased the number of high‐affinity sites for [3H]‐dopamine without affecting dissociation constant. However, the PAMs did potentiate [3H]‐ dopamine binding at D3 receptors. As a result, these compounds have a paramount importance in the treatment of hypodopaminergic function.^81^, ^82^

The treatment of schizophrenia with the currently available medications is often worrisome because of the challenge in obtaining a better therapeutic effect without serious side effects. As a result, allosteric modulators are a good candidate to overcome these problems, at least in part. Indeed, SB269652 is the first negative allosteric modulator of dopamine D3 receptors^82^, ^83^ that initiate researchers to discovery new D3 allosteric modulators.^84^ The development of optimized analogs has led to purer allosteric modulators and potent bitopic modulators. As a result, it is worth the trouble to design optimized derivatives of SB269652 to explore their efficacy in clinic.^85^, ^86^, ^87^

## 3 CONCLUSION AND FUTURE DIRECTIONS

The exploration of allosteric modulation of dopamine receptors stands as a promising frontier in drug discovery, especially for addressing central nervous system disorders. Conventional drug development strategies targeting orthosteric ligands have grappled with issues of selectivity and side effects. Allosteric modulators, categorized as positive allosteric modulators (PAMs), negative allosteric modulators (NAMs), and silent allosteric modulators (SAMs), present a sophisticated and adaptable approach to finely tune receptor function. The pivotal role of allosteric modulators lies in their capacity to induce conformational changes in G protein‐coupled receptors (GPCRs), including dopamine receptors, leading to a spectrum of pharmacological effects. This modulation offers advantages such as enhanced drug selectivity, diminished side effects, and improved therapeutic outcomes. The sensitivity of allosteric modulators to alterations in protein conformation not only propels drug discovery but also contributes significantly to comprehending receptor mechanisms.

Exploring biased allosteric modulators to selectively activate desired signaling pathways of dopamine receptors holds promise for developing drugs with specific therapeutic effects and minimized side effects. Advances in structural biology, including cryo‐electron microscopy and X‐ray crystallography, offer crucial insights into dopamine receptor three‐dimensional structures, aiding rational drug design for more potent and selective allosteric modulators. Investigating combinations of allosteric modulators with traditional dopamine agents or other psychiatric medications may enhance treatment outcomes. Understanding interactions between dopamine receptors and other neurotransmitter systems could facilitate the development of allosteric modulators targeting multiple receptors for synergistic therapeutic effects. Neuroimaging techniques like PET and fMRI can validate in vivo efficacy and inform clinical translation. Tailoring allosteric modulators to specific neurological disorders (e.g., schizophrenia, Parkinson's, addiction) based on unique pathophysiology's may yield more targeted therapeutic strategies.

In general, allosteric modulation of dopamine receptors emerges as an intriguing strategy for drug development, presenting novel therapeutic avenues while addressing challenges inherent in traditional approaches. Ongoing research in this domain holds the promise of uncovering additional allosteric modulators, paving the way for innovative solutions in the treatment of central nervous system disorders.

## AUTHOR CONTRIBUTIONS

**Fentaw Girmaw**: Conceptualization; data curation; formal analysis; funding acquisition; investigation; methodology; project administration; resources; software; supervision; validation; visualization; writing—original draft; writing—review and editing.

## CONFLICT OF INTEREST STATEMENT

There is no conflict of interest.

## ETHICS STATEMENT

The authors have nothing to report.

## TRANSPARENCY STATEMENT

Fentaw Girmaw affirms that this manuscript is an honest, accurate, and transparent account of the study being reported; that no important aspects of the study have been omitted.

## Data Availability

The authors confirm that the data supporting the findings of this study are available within the article [and/or] its supplementary materials.
